# Cooperation and interplay between base and nucleotide excision repair pathways: From DNA lesions to proteins

**DOI:** 10.1590/1678-4685-GMB-2019-0104

**Published:** 2020-03-02

**Authors:** Namrata Kumar, Natália C. Moreno, Bruno C. Feltes, Carlos FM Menck, Bennett Van Houten

**Affiliations:** ^1^ University of Pittsburgh University of Pittsburgh School of Medicine Department of Microbiology and Molecular Genetics PittsburghPA USA University of Pittsburgh, School of Medicine, Department of Microbiology and Molecular Genetics, Pittsburgh, PA, USA.; ^2^ University of Pittsburgh University of Pittsburgh UPMC Hillman Cancer Center PittsburghPA USA University of Pittsburgh, UPMC Hillman Cancer Center, Pittsburgh, PA, USA.; ^3^ Universidade de São Paulo Universidade de São Paulo Instituto de Ciências Biomédicas Departamento de Microbiologia São PauloSP Brazil Universidade de São Paulo, Instituto de Ciências Biomédicas, Departamento de Microbiologia, São Paulo, SP, Brazil.; ^4^ Universidade Federal do Rio Grande do Sul Universidade Federal do Rio Grande do Sul Instituto de Informática Porto AlegreRS Brazil Universidade Federal do Rio Grande do Sul, Instituto de Informática, Porto Alegre, RS, Brazil.; ^5^ University of Pittsburgh University of Pittsburgh School of Medicine Department of Pharmacology and Chemical Biology PittsburghPA USA University of Pittsburgh, School of Medicine, Department of Pharmacology and Chemical Biology, Pittsburgh, PA, USA.

**Keywords:** Base excision repair, nucleotide excision repair, DNA damage, protein oxidation, UVA light

## Abstract

Base and nucleotide excision repair (BER and NER) pathways are normally associated with removal of specific types of DNA damage: small base modifications (such as those induced by DNA oxidation) and bulky DNA lesions (such as those induced by ultraviolet or chemical carcinogens), respectively. However, growing evidence indicates that this scenario is much more complex and these pathways exchange proteins and cooperate with each other in the repair of specific lesions. In this review, we highlight studies discussing the involvement of NER in the repair of DNA damage induced by oxidative stress, and BER participating in the removal of bulky adducts on DNA. Adding to this complexity, UVA light experiments revealed that oxidative stress also causes protein oxidation, directly affecting proteins involved in both NER and BER. This reduces the cell’s ability to repair DNA damage with deleterious implications to the cells, such as mutagenesis and cell death, and to the organisms, such as cancer and aging. Finally, an interactome of NER and BER proteins is presented, showing the strong connection between these pathways, indicating that further investigation may reveal new functions shared by them, and their cooperation in maintaining genome stability.

## Introduction

It has been suggested that every cell in our body suffers tens of thousands of lesions per day (Lindahl and Barnes, 2000; Tubbs and Nussenzweig, 2017), which if left unrepaired, may lead to mutations, genome instability and cancer. DNA damage can occur from exogenous sources like ultraviolet (UV) light, ionizing radiation (IR), and chemical exposure from pollutants in the air and water. Genomic damage can also be produced from endogenous processes such as replication errors or reactive oxygen species (ROS) from mitochondria or inflammation. Since DNA damage occurs continuously in all living systems, organisms have evolved efficient systems to ameliorate the harmful effects of environmental genotoxicants. Depending on the type of lesion formed in the DNA, six major repair pathways play a key role in maintaining genome stability, these include: direct reversal, base excision repair (BER), nucleotide excision repair (NER), mismatch repair (MMR), recombination with two major sub-pathways: homologous recombination (HR) and non-homologous end joining (NHEJ), and interstrand cross-link (ICL) repair which combines features of several pathways including NER and recombination, and is controlled by a wide range of proteins. There are also several dedicated translesion DNA polymerases that allow the replication machinery to bypass specific lesions, at the expense of lowered fidelity (Goodman, 2002). Furthermore, key signaling pathways are controlled by transcription factors like p53 and DNA kinases including ATM, ATR and DNA-PK. Although these pathways have been described to work independently, there are indications that in fact these may interact, in a network for maintaining genome protection. This review emphasizes on the interplay between some of the proteins involved in either NER or BER. As for other DNA repair pathways, proteins that participate in NER and BER are also subject to injury, mainly by oxidation, an effect that has been little explored. However, this effect, initially described as an effect of UVA on cells, may interfere on the cells’ ability to process DNA damage, adding a new level of complexity in the analysis of NER and BER interplay, as discussed below.

NER consists of a group of proteins that participate in the repair of lesions that cause significant helical distortion in the DNA structure, such as those induced by UV light, environmental mutagens like polycyclic aromatic hydrocarbons (PAHs) and certain chemotherapeutic agents like cisplatin (Wood, 1999; Scharer, 2013). UVC (254 nm) produces mainly cyclobutane pyrimidine dimers (CPD) and pyrimidine (6-4) pyrimidone photoproducts (6-4PP), while cisplatin forms intra- or interstrand Pt-adducts. Interestingly, longer wavelengths UVB (280-320 nm) and UVA (320-400 nm), which penetrate the earth’s atmosphere, can produce a spectrum of lesions including photoproducts and oxidized bases, removed by NER and as well as BER. NER includes two sub-pathways: global genome NER (GG-NER) and transcription-coupled NER (TC-NER). GG-NER operates in the entire genome, including untranscribed regions and silent chromatin, while TC-NER recognizes and repairs bulky DNA lesions in the transcribed DNA strands of active genes only. In GG-NER, XPC-RAD23B acts as the initial damage recognition factor by recognizing destabilized DNA (Sugasawa *et al.*, 1998). UV-DDB is part of a Cul4-RBX1 ubiquitin ligase, which upon UV radiation ubiquitinates DDB2, histones and XPC. While ubiquitinated DDB2 is degraded, XPC shows an elevated DNA binding activity. During the damage verification step of GG-NER, the transcription factor TFIIH is recruited by XPC-RAD23B protein (Sugasawa *et al.*, 2005; Kapetanaki *et al.*, 2006; Wang *et al.*, 2006). TFIIH consists of 10 subunits, including the helicases XPB and XPD that are responsible for opening up the DNA around the lesion (Evans *et al.*, 1997). XPD binding to the lesion facilitates the recruitment of the pre-incision complex (XPA, RPA, XPG) (Wakasugi and Sancar, 1998; Volker *et al.*, 2001; Riedl *et al.*, 2003). Once the second endonuclease ERCC1-XPG is recruited, dual incision by XPG and XPF is initiated and the excision product is released along with TFIIH (Kemp *et al.*, 2012). DNA polymerase (δ/ ε) and ligase I then repair and ligate the gap (Shivji *et al.*, 1995). TC-NER, on the other hand, is triggered by stalled RNA polymerase at a DNA lesion during transcription, causing the Cockayne syndrome proteins (CSB and CSA), and other lesion accessory proteins (UVSSA, XAB2, and HMGN1) to be recruited at the lesion site. With the subsequent recruitment of TFIIH, TC-NER converges with the GG-NER at this step (Fousteri and Mullenders, 2008). Mutations in these NER proteins impair the ability to repair UV damage, causing autosomal recessive disorders including xeroderma pigmentosum (XP) (mutations in XPA-G, XPV) characterized by extreme sensitivity to sunlight and increased risk to skin cancer in exposed areas. About 20-30% of these patients also develop neurodegeneration. Also mutations in CSA and CSB, affecting only TC-NER, result in Cockayne syndrome (CS), with patients presenting developmental impairment and neurodegeneration, related to premature aging (Marteijn *et al.*, 2014; Menck and Munford, 2014; Karikkineth *et al.*, 2017).

**Figure 1:**
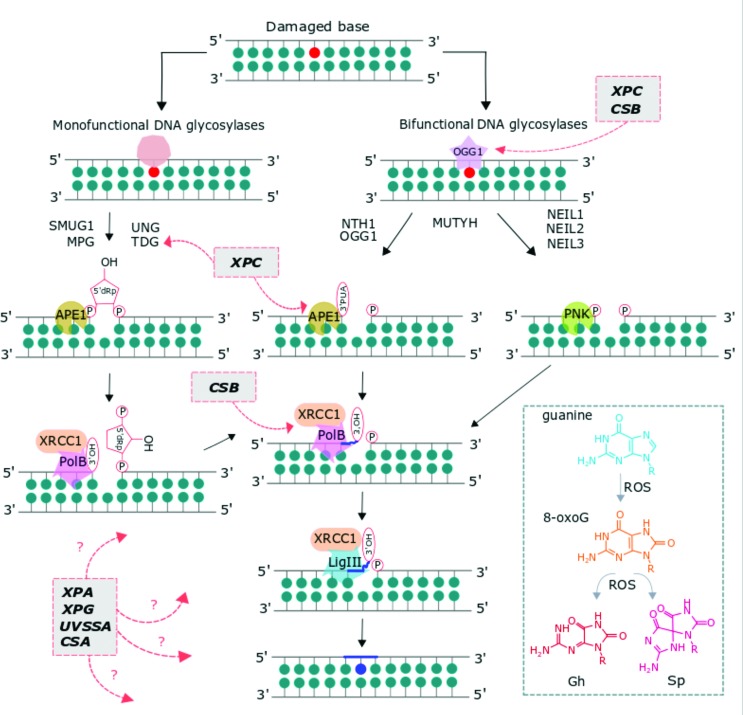
Mono- and bi-functional DNA glycosylase-initiated short-patch base excision repair (BER) in mammalian cells. The process consists of these main steps: Excision of the base lesion, incision by an AP endonuclease, end processing, gap filling and ligation. Insert shows the common oxidative lesions repaired by BER: 8-oxoguanine (8-oxoG), guanidinohydantoin (Gh), spiroiminodihydantoin (Sp). Grey boxes (red dashed outline) indicate the involvement of NER (XPA, XPC, XPG, CSA, CSB and UVSSA) proteins in BER.

BER is a dedicated pathway that removes a wide range of chemically altered bases (Svilar *et al.*, 2011; Wallace, 2014; Bauer *et al.*, 2015; Thapar and Demple, 2017; Whitaker *et al.*, 2017) (Figure 1). This type of damage typically results from spontaneous reactions in the cells (deamination, oxidation and methylation), metabolic by-products (ROS) and exogenous sources like alkylating agents (methyl methane sulfonate), ionization radiation (IR), X-rays, and pollutants, including cigarette smoke. Due to its redox potential, guanine is the most susceptible base to oxidation, forming mainly 8-oxoguanine (8-oxoG). This lesion is highly mutagenic and if not repaired, can pair with adenine, causing a G:C to T:A transversion. One of the earliest steps in the repair of base lesions is lesion recognition and removal by DNA glycosylase. In the case of 8-oxoG, this is mediated by a dual functional glycosylase, 8-oxoG glycosylase (OGG1) which first removes the damage through hydrolysis of the glycosidic bond, creating an apurinic/apyrimidinic (AP). This abasic site is acted on by a weak lyase activity of OGG1 causing cleavage 3’ to the abasic site. OGG1 has higher affinity for the abasic site and is therefore product inhibited, and needs the action of an AP endonuclease (APE1), to help turn the enzyme over and cleave, leaving a 5’ deoxyribose-phosphate moiety generating a one base pair gap (Hill *et al.*, 2001). Some models of BER show poly(ADP)-ribose polymerase (PARP1) activation during this transient nick and gap phase, which, through the production of poly(ADP)ribose, helps recruit the remaining DNA repair factors, XRCC1, a scaffold protein, DNA polymerase beta and DNA ligase I. During repair this gap is filled in by DNA polymerase beta, and ligated by DNA ligase I or III.

While both BER and NER pathways have been conventionally associated with specific substrates, growing evidence shows a significant cooperation between these two repair mechanisms, and has recently been reviewed (Melis *et al.*, 2013; Limpose *et al.*, 2017; Shafirovich and Geacintov, 2017). The relevance of this potential interaction includes the fact that NER deficient (XP and CS) patients may develop developmental and neurological symptoms, related to premature aging, that can be due to endogenous lesions, such as DNA damage induced by oxidation, which are normally considered substrates for BER. Thus, understanding BER and NER interplay may help us to better understand the causes for the symptoms of premature aging found in these patients and even for the normal aging process. In fact, certain types of DNA damaging agents can result in a spectrum of DNA lesions that are handled by different DNA repair pathways, thus it is expected that proteins and enzymes from different pathways may cooperate to remove specific types of DNA damage. Moreover, these enzymes may also be injured by oxidation affecting, as shown by UVA irradiation, a component that also generates a mixture of DNA lesions that involves both NER and BER. This review discusses some of the more recent advances made in understanding the interplay between these two pathways by discussing specific DNA lesions and the proteins that recognize and remove them. The additional effects of oxidation of proteins related to these pathways by UVA exposure also interfere with the cells’ capacity to process the different types of DNA lesions, with possible biological consequences such as carcinogenesis. This is also reviewed, focusing on the effects on NER and BER, and proteins that act on both pathways.

## Oxidatively generated base damage recognized by NER pathway

### Cooperative interactions in processing 8-oxoG

A number of base modifications are recognized by the BER pathway (Figure 1), but one of the most common and well-studied lesions is 8-oxoguanine (8-oxoG). As described above, 8-oxoG is processed by OGG1 through BER, although recent studies show that other proteins and sub-pathways may partner in this process. One of the earliest experiments suggesting an involvement of NER proteins in the repair of oxidized base damage was an *in vitro* study from the Sancar laboratory. They showed that cell free extracts from human cell lines either lacking or containing mutated NER proteins (XP-A, XP-B, XP-C, XP-D, XP-F and XP-G) had markedly reduced ability to excise two major oxidized base damage, 8-oxoG and thymine glycol (TG), as part of an excision oligonucleotide consistent with NER (Reardon *et al.*, 1997). They went on to show complete NER system reconstituted with purified XPA, RPA, TFIIH (containing XPB and XPD), XPC-HHR23B, XPG, and ERCC1-XPF proteins, were necessary and sufficient to excise 8-oxoG or TG. While these studies indicated that NER proteins are capable of acting on two common oxidized bases, whether NER proteins had a direct role in BER by interacting with BER proteins or intermediates was uncertain. The authors suggested that perhaps NER is a relatively slow back-up system for BER.

D’Errico *et al.* (2006) provided the first direct evidence that XPC plays a role in the protection against oxidative stress. They demonstrated that keratinocytes and fibroblasts with mutations in XPC were extremely sensitive to potassium bromate and ionizing radiation. Using LC/MS and HPLC-ED, they were able to show the accumulation of 8,5’-cyclopurine 2’-deoxynucleosides and slow removal of 8-oxoG and 8-oxoA, respectively, in cells lacking XPC. Biochemical assays with purified proteins showed stimulation of DNA glycosylase OGG1 by XPC-HR23B and western blots showed that purified XPC-HR23B interacted directly with OGG1. Unlike XPC, at the concentrations surveyed, XPA was not capable of stimulating OGG1. This study indicates that XPC-HR23B facilitated recognition of 8-oxoG in an OGG1-dependent BER. It is interesting to note that XP-C patients, in addition to high skin cancer rates, also have a higher incidence of internal cancer development (Giglia *et al.*, 1998; Hollander *et al.*, 2005; Sarasin *et al.*, 2019). Thus, reduced kinetics of oxidatively generated DNA damage might be a major contributor to these internal cancers. Moreover, oxidatively generated base damage are also associated with increased risk of neurodegenerative diseases (Chen *et al.*, 2012; Liu *et al.*, 2017). While XP-A, XP-B, XP-D and XP-G patients may show neurodegeneration symptoms, XP-C patients show no signs of neurological defects. Thus, it is possible that XPC might be acting as a cofactor in the repair process, therefore its loss alone does not display major effects. In a separate study by Kassam and Rainbow (2007), methylene blue plus visible light (photoactivated MB, which generates singlet oxygen) was used to produce 8-oxoG in an adenovirus-encoded β-galactosidase (β-gal) reporter gene, and a host cell reactivation (HCR) assay was used to demonstrate that human cells deficient in XPC showed lower HCR as compared to WT cells, supporting a role for XPC in the processing of 8-oxoG (Kassam and Rainbow, 2007). Similarly, XP-A and XP-C NER deficient cells were found to be more sensitive to photoactivated MB, compared to NER proficient cells (Berra *et al.*, 2013). Problems dealing with the oxidized base damage, in XP-A and XP-C cells, were confirmed with observations of cell cycle delay (increased G2/M arrest) and genotoxic stress (H2AX phosphorylation). These results confirm NER proteins participate in the processing of oxidatively generated base damage, although which type of lesion (including 8-oxoG) is involved was not clear.

In order to better understand the potential roles of XPA and XPC in the removal of oxidized bases, Parlanti *et al.* (2012) went on to study the rates of 8-oxoG removal, as measured by HPLC–ED, in mouse embryo fibroblasts (MEFs) derived from NER (Csb^m/m^, Csa^-/-^ Xpa^-/-^ Xpc ^-/-^ and combinations of these) and/or Ogg1^-/-^ deficient mouse mutants following treatment with the oxidizing agent, potassium bromate. While Ogg1^-/-^ deficient mutant cells displayed a dramatic deficiency in the rate of 8-oxoG removal, NER deficient mutants (Csb^m/m^, Csa^-/-^ Xpa^-/-^ Xpc ^-/-^) also displayed reduced rates of removal as compared to WT MEFs. Furthermore, Csb^-/-^ Xpa^-/-^ and Csb^-/-^ Xpc^-/-^ double mutants were more deficient in repair as compared to the single mutants, and very similar to the deficiency observed in Ogg1^-/-^ MEFs. On the other hand, Xpc^-/-^ Xpa^-/-^ double mutant did not show slower repair kinetics as compared to the single mutant MEFs, suggesting that XPC and XPA function through the same pathway, while CSB is OGG1-dependent, but XPA/XPC independent. These mouse experiments were confirmed in human XP-A primary fibroblasts that were more sensitive to potassium bromate as compared to WT fibroblasts. Furthermore, SV40-transformed XP-A deficient cell line (XP12SV40), in which OGG1 was knocked down with siRNA, showed slower 8-oxoG repair kinetics than either the XP-A cells alone or when XPC was knocked down. Whether this enhanced repair of 8-oxoG through the action of XPA, XPC, and CSB is mediated through canonical BER is unclear. Why XPA did not stimulate OGG1 activity in their previous study, but a deficiency in XPA showed a slower rate of 8-oxoG remains to be reconciled. Also, the involvement of these proteins could vary in the context of chromatin accessibility. Finally, it is interesting to note that the Xpa^-/-^/Xpc^-/-^ and Csb^m/m^/Ogg1^-/-^ double mutant mice are viable and do not show evidence for neurodegeneration (Friedberg and Meira, 2006; Laposa *et al.*, 2007). 

XP-G deficient cells were also found to be sensitive to the treatment with photoactivated MB, indicating that XPG protein, and thus NER, participate in the processing of oxidized base damage (Soltys *et al.*, 2013). This was observed for cells from a severely affected patient, with neurological problems, carrying an *XPG* mutation that completely abrogates the protein. The increased sensitivity was also confirmed by HCR of plasmids treated with photoactivated MB. Interestingly, two different *XPG* missense alleles, from patients with no neurological symptoms (but with XP typical increased frequency of skin tumors), showed sensitivity to UV-light induced DNA damage, but not to oxidized base damage induced by singlet oxygen. These results indicate that XPG protein might participate on the removal of UV-induced lesions by NER, with an independent function for oxidatively generated base damage, and defects on this latter function is in fact relevant for the induction of neurological symptoms in XP-G patients.

### Cellular imaging of 8-oxoG processing involving CSB and XPC

Menoni *et al.* (2012) used a novel imaging tool to study the role of XPC and CSB in the repair of oxidized base damage in living cell. By using a photosensitizer Ro 19-8022 and 405 nm laser light, they were able to generate localized oxidized base damage in specific regions of the nucleus. XPC-GFP and CSB-GFP both were seen to be recruited to the sites of damage. CSB appeared to be recruited faster than XPC, possibly due to different intrinsic mobility or chromatin binding properties. Indeed, they reported that CSB was prominently recruited in the nucleolus (possibly due to high transcription activity) and XPC accumulated more densely in the heterochromatic region, consistent with their roles in TC-NER and GG-NER of UV-induced photoproducts, respectively. Interestingly, they reported, but did not show the data, that neither XPB nor XPA was recruited to the damage site even after 5-10 minutes of damage induction. These data suggesting that CSB and XPC recruitment was independent of subsequent steps in NER is in contrast to the work by Parlanti *et al.* (2012) who showed that both XPA and XPC might facilitate 8-oxoG removal.

In a more recent study by Vermeulen’s group, the role of CSB in 8-oxoG repair was further elaborated (Menoni *et al.*, 2018). Using the live-cell imaging approach described above, it was shown that OGG1 recruitment to the damage site was independent of CSB, but the recruitment of the BER scaffolding protein XRCC1 was stimulated by CSB in a transcription-dependent manner. It is possible that as a chromatin remodeler, CSB helps XRCC1 loading under certain circumstances, perhaps in transcribed genes or at specific genomic regions that are not accessible to the downstream BER proteins.

### Comet-FISH assay reveals an involvement of XPA, CSB, and UVSSA in TCR of 8-oxoG

As noted above, the role of XPA in the processing of 8-oxoG adducts has been controversial, and contrasting studies have been published. In an elegant tour-de-force study, Guo *et al.* (2013) combined a single-cell electrophoresis (Comet assay) with fluorescence *in situ* hybridization (FISH) and established the involvement of XPA and CSB preferentially in transcription-coupled 8-oxoG removal. For these experiments, 5’- and 3’-ends of the ATM gene were labelled with different fluorescent probes. The increase in the distance between the probes after damage was an indication of single strand breaks. The repair rates of transcribed and non-transcribed strands in CS-B and XP-A cells were similar, indicating that they played a role in TCR of 8-oxoG. They also showed that elongating RNAP II and UVSSA were necessary for this process consistent with TCR. The authors speculated that after initial recognition and incision by OGG1 and APE1, the single stranded DNA formed causes a block to transcription, recruiting the TCR proteins to continue repair. This model is consistent with the work by Vermeulen’s group cited above. XPC, since it is involved in GG-NER, was not investigated in this study.

### XPC also stimulates a thymine specific DNA glycosylase

Spontaneous deamination of C or 5-methylC creates dU-G and T-G mismatches which are processed by uracil DNA glycosylase (UDG) family and thymine DNA glycosylase (TDG), respectively. XPC-HR23B was shown to stimulate TDG activity in an in vitro nicking assay (Shimizu *et al.*, 2003). While XPC, itself, did not have any effect on nicking the G/T mismatch oligonucleotide, it stimulated TDG activity in a dose dependent manner, probably promoting enzymatic turnover of TDG. XPC also stimulates OGG1 binding to damaged DNA, and a weak interaction between the proteins was obtained from far western analysis (Parlanti *et al.*, 2012). Finally, Melo *et al.* (2016) showed a correlation between XPC deficiency and OGG1/ APE1 expression levels, and a physical interaction between XPC and APE1 using co-immunoprecipitation. These studies are summarized in Tables 1 and 2.

**Table 1:** Oxidative lesions recognized by NER factors.

Lesions	Protein involved	References
8-oxoG and TG^*^	NER proteins	Reardon *et al.* (1997)
8-oxoG	XPC-CSB (TC-BER)	Menoni *et al.* (2012)
8-oxoG	CSB (TC-BER)	Menoni *et al.* (2018)
8-oxoG	XPA, CSB and UVSSA	Guo *et al.* (2013)
8-oxoG	XPC/XPA	Parlanti *et al.* (2012)
Guanine lesions	NER proteins	Shafirovich *et al.* (2019)

* 8-oxoG and thymine glycol

**Table 2:** Protein interactions between BER and NER.

Protein-protein interaction	References
XPC-HR23B and TDG	Shimizu *et al.* (2003)
XPC and OGG1	D’Errico *et al.* (2006)
XPC and APE1/OGG1	Melo *et al.* (2016)

### Oxidized guanine lesions are excised more efficiently by competing BER than NER pathways

The base damage, 8-oxoG is susceptible to further oxidation, leading to the formation of spiroiminodihydantoin (Sp) and 5-guanidinohydantoin (Gh), which are recognized by the DNA glycosylase NEIL1 (Luo *et al.*, 2000; Niles *et al.*, 2001; Hailer *et al.*, 2005; Krishnamurthy *et al.*, 2008; Zhao *et al.*, 2010).

Very recently, Shafirovich *et al.* (2019) examined the excision of these lesions in intact human cells and the relative contribution of BER and NER in the processing of these lesions. In this study, an internally labelled hairpin substrate containing these lesions were transfected into HeLa cells. DNA was isolated at different time points and run on a PAGE gel. The BER activity was determined by the presence of a 65nt incision product, while the presence of a 24-30nt excision product indicated NER activity. The hairpins with both Gh and Sp lesions exhibited BER, as well as NER activity, suggesting a competition between these two pathways in repair. Addition of unlabeled hairpin with a known BER substrate 5-OHU caused significant reduction in the BER product, but an increase in the NER product. This suggests that the participation of these two pathways depends on the local concentration of the recognition factors that recognize and bind to the same lesions in a competitive manner.

## Bulky DNA lesions recognized by BER pathway

### BER protects cells against Pt-adducts

Platinum-based drugs are most widely used for the treatment of cancer (Kelland, 2007). The three approved drugs for treatment are: cisplatin, oxaliplatin and carboplatin. These drugs form platinum adducts by either covalently linking two nucleotide residues on the same DNA strand (intrastrand crosslink) or from opposite strands (interstrand crosslink- ICL). Left unrepaired, ICLs cause cytotoxicity by blocking transcription and replication (Huang and Li, 2013). Although this damage is largely repaired by NER, Kim *et al.* (2015) showed that APE1 protective role against damage caused by ICLs. Using a slot-blot assay and an antibody against 1,2-Pt-(GpG) DNA adducts, they demonstrated that reducing the expression of APE1 by siRNA inhibited the repair of cisplatin adducts. This inhibition was restored by adding back APE1 with repair activity, but not the redox signaling function. Furthermore, altering APE1 expression affected the expression levels of two NER proteins, RPA and XPA, suggesting an interaction between these two pathways. However, it should be pointed out that cisplatin exposure is also known to induce ROS production (Marullo *et al.*, 2013), therefore explaining the involvement of BER proteins in the repair process, and APE1 expression may also help to protect repair proteins from oxidation (see below). Therefore, more studies are required to unravel the exact role and interplay between BER and NER proteins in the repair process of cisplatin induced DNA damage.

Slyskova *et al.* (2018) recently used a CRISPR/Cas9 screen to determine which proteins and pathways are involved in the repair of oxaliplatin and cisplatin induced adducts. These drugs covalently bind to DNA and form crosslinks, mainly Pt-GpG (60–65%) and Pt-ApG (25–30%), along with monoadducts (2%). They showed that the proteins involved in TC-NER and/or BER were essential in protecting cells against the cytotoxicity of oxaliplatin and cisplatin. Using fluorescence recovery after photobleaching (FRAP), they showed evidence for the recruitment of CSA, CSB, and XRCC1 at localized ICLs, generated by 8-methoxypsoralen+UVA, in living cells. Additionally, the recruitment of these proteins was found to be transcription dependent, as the recruitment was suppressed by blocking RNAP elongation using flavopiridol. Finally, by knocking down OGG1 and XPA, they were able to determine that the recruitment of XRCC1 was BER dependent, but NER independent. Measurement of H_2_DCFDA fluorescence was used to validate that platinum drugs generate oxidatively generated damage, necessitating the presence of BER proteins. The oxidized base damage could cause an accumulation of BER intermediates like abasic sites and single-strand breaks that contribute to the transcription block, along with the ICLs. These data might help explain the presence of TC-NER proteins, CSA and CSB, but not GG-NER proteins, XPC or DDB2. Apart from acting on the adducts directly, it is possible that CSB is recruited to these oxidized base damage in a transcription- dependent manner, to recruit XRCC1, as described previously (Menoni *et al.*, 2012, 2018).

### DNA glycosylase NEIL1 binds and excises psoralen- induced monoadducts and interstrand crosslinks

Using a combination of excision assays, cell survival assays, and *in vitro* BER assay, Couve-Privat *et al.* (2007) showed that NEIL1 and APE1 deficient cells are sensitive to 8MOP+UVA. There was no further increase in sensitivity when both these proteins were depleted together, suggesting that they function via the same pathway. Additionally, these proteins are able to excise psoralen monoadducts, but not ICLs, in duplex DNA (Couve-Privat *et al.*, 2007). Specifically, NEIL1 cleaves the monoadduct generating 3’-phosphate termini, that is removed by APE1. Finally, by reconstituting BER *in vitro,* they show that NEIL1 and APE1 can repair psoralen monoadducts in a pol-β dependent manner. To further elucidate the role of NEIL1 in the repair of psoralen ICLs, the group used a three-stranded DNA structure with an unhooked ICL, which is a physiological representative of an ICL lesion, after being acted upon by endonucleases. They show that NEIL1 could excise this substrate and catalyze an *in vitro* BER reaction, indicating multiple modes of action of NEIL1 in psoralen adduct repair (Couve *et al.*, 2009). Another study by the same group looked into the role of NEIL1 and NEIL3 in the repair of ICL repair intermediates like the three- and four-stranded DNA structures, generated via FANCM mediated replication fork bypass and demonstrated that both glycosylases participate in the repair (Martin *et al.*, 2017). In a contrasting study by McNeill *et al.* (2013) it was shown that NEIL1 was recruited specifically to ICLs but not monoadducts. In living cells, when treated with trioxsalen, NEIL1 recruitment was not affected in the presence of an antioxidant, N-acetyl-L-cysteine (NAC), indicating different mechanisms of recruitment to oxidative damage and ICLs respectively. Interestingly, NEIL1 was recruited and dispersed within 8 minutes post irradiation, while XPC was seen until 60 minutes after damage induction, suggesting that the glycosylase was not being recruited as part of the XPC complex. Moreover, NEIL1 deficient cells were resistant to psoralen + UVA damage, and had a faster rate of psoralen removal, hinting on a negative role of NEIL1 in the repair of ICLs. The authors theorize that the contradicting results could be a result of variation in techniques or using trioxsalen versus 8-methoxypsoralen for damage induction. While 8-MOP produces about 20% monoadducts, trioxsalen generates < 2%. Future studies are required to understand these discrepancies in more detail. These studies are summarized in Table 3.

**Table 3:** Bulky lesions recognized by BER.

Lesions	Protein involved	References
Pt-adducts	APE1	Kim *et al.* (2015)
Pt-adducts	OGG1/XRCC1	Slyskova *et al.* (2018)
ICLs	NEIL1	McNeill *et al.* (2013)
		Couvé-Privat *et al.* (2007)
		Couvé *et al.* (2009)
		Martin *et al.* (2017)

## Repair proteins as target of UVA light-induced oxidative stress:

### UVA light forms a mixture of photoproducts and oxidatively generated base damage:

Ultraviolet (UV) light is a well-known DNA damaging agent, and most of the organisms on this planet are exposed to it via sunlight, with important pathophysiological consequences such as skin carcinogenesis and photoaging. While the ozone layer shields the surface of the earth from harmful UVC (100-280 nm) light, more than 90% of UVB (280-320 nm) and UVA (320-400 nm) reach the earth’s surface. UVA light plays an important role in sunlight-induced DNA damage, as it corresponds to 95% of sunlight UV component, and penetrates deeper in the human skin because of its longer wavelength.

UVA light induces a mixture of different types of DNA lesions, including photoproducts and oxidized bases, which are then repaired by both NER and BER, providing an interesting model to investigate the DNA repair capacity of these pathways (Ravanat *et al.*, 2001; Sage *et al.*, 2012; Schuch *et al.*, 2017). Pyrimidine dimers, such as CPDs and 6-4 PPs, are formed through direct photon absorption by DNA bases (Schuch *et al.*, 2009; Cortat *et al.*, 2013). Evidence for the role of UVA light in causing direct DNA damage was demonstrated as early as in 1973, by observing the formation of CPDs in the genome of *Escherichia coli (*Tyrrell*,* 1973*)*. More recently, the biological relevance of pyrimidine dimers (CPDs) induced by UVA was observed in Chinese hamster cells and in human skin (Douki *et al.*, 2003; Mouret *et al.*, 2006). Although 6-4PPs (t1_/2_ ~2-4 hrs) are removed by NER at a significantly faster rate compared to CPDs (t_1/2_ ~ 24 hrs) , they were also detected upon UVA light exposure in DNA repair deficient cell models (Schuch *et al.*, 2009; Cortat *et al.*, 2013). It is important to mention that 6-4 PP undergo Dewar isomerization, to form a new photoproduct, which is repaired by NER. In fact, upon exposure to sunlight, UVA radiation converts the 6-4 PP into Dewar PP (Clingen *et al.*, 1995; Perdiz *et al.*, 2000; Douki *et al.*, 2003; Douki and Sage, 2016).

UVA light also induces DNA damage by mechanisms that involve oxidative stress, generated as a result of irradiation. Intracellular ROS can be generated through photosensitization reactions caused by endogenous chromophores absorbing UVA-light, including DNA, urocanic acid, pophyrins, flavins, melanin and their precursors and metabolites (Emri *et al.*, 2018). These photosensitized molecules, normally in the triplet state, can either react directly with DNA (type I reaction) or transfer their energy to molecular oxygen, to form ^1^O_2_ and subsequently generate ROS (type II reaction). In both cases, the result may be DNA oxidation (Di Mascio *et al.*, 1990; Halliwell and Aruoma, 1991; Evans *et al.*, 2004). ROS may also be generated as a delayed response to irradiation, probably due to the activation of cellular enzymes, such as NADPH oxidases and cyclooxygenases (Valencia and Kochevar, 2008; Birch-Machin and Swalwell, 2010). UVA-induced ROS can generate a variety of modifications, including 8-oxoG, abasic sites, single and double strand breaks and crosslinks (Cadet *et al.*, 2005; Schuch *et al.*, 2017).

UVA induces both direct and indirect DNA damage, repaired by NER and BER, respectively. Mutational effects by UVA are typically due to lesions induced by direct DNA absorption (pyrimidine dimers). Most of the published studies have reported C > T changes at dipyrimidine sites (Robert *et al.*, 1996; Ikehata *et al.*, 2003; Agar *et al.*, 2004; Kappes *et al.*, 2006), which is similar to UVC and UVB induced mutagenesis (Brash *et al.*, 1987; Douki *et al.*, 2003; Kappes *et al.*, 2006; Herman *et al.*, 2014). Interestingly, this type of mutation has been detected in nonmelanoma (Giglia-Mari and Sarasin, 2003), as well as, melanoma skin cancers (Greenman *et al.*, 2007; Pleasance *et al.*, 2010). However, the participation of UVA light-induced oxidative stress in these mutagenic and damaging processes cannot be completely ruled out. Cells have a broad array of antioxidant mechanisms, which provide the initial defense to minimize the oxidation of proteins, DNA and other biomolecules. Human skin has elaborate enzymatic and non-enzymatic defenses against ROS, such as the superoxide dismutase (SOD), catalase (CAT), and glutathione (GSH)/glutathione peroxidase systems. The transcription factor, Nrf2 (NF-E2-related factor 2) coordinates the activation of several genes whose products participate in the cellular response to the oxidation of biomolecules. Further, studies have shown that Nrf2 plays a protective role in keratinocytes and fibroblasts against the damaging effects of UVA-induced DNA lesions (Hirota *et al.*, 2005; Tian *et al.*, 2011).

### UVA light interferes with DNA repair as a consequence of protein oxidation

Apart from the antioxidant systems, DNA repair mechanisms act as a protection barrier against UVA-induced lesions. As commented earlier, NER generally repairs lesions that cause significant distortions in the DNA molecule, such as CPDs and 6-4 PPs, whereas BER repairs ROS-induced small base covalent modifications. Therefore, interplay between these two processes would be important to deal with the variety of damage induced after UVA-irradiation. Work on UVA has revealed that proteins and lipids are also affected by ROS, and, interestingly, proteins involved in DNA repair are highly sensitive to oxidation. Studies show that UVA-light in the presence of photosensitizers caused extensive protein oxidation, affecting DNA damage removal by NER (Peacock *et al.*, 2014), as well as BER (Gueranger *et al.*, 2014). Thus, protein oxidation may be a direct consequence of UVA irradiation increasing the mutation risk by sunlight (McAdam *et al.*, 2016). Confirming these observations, studies from our lab showed that protein oxidation by UVA irradiation also affects the ability of human cells to replicate their genetic material, probably due to translesion synthesis (TLS) and NER being affected in irradiated XP-V cells (Moreno *et al.*, 2019a b). Curiously, previous work reported that UVA-induced singlet oxygen leads to DNA replication arrest independently of cell cycle checkpoints activation, probably due to a transient decrease of dNTP pool, probably not related to the oxidation of DNA repair proteins (Graindorge *et al.*, 2015). This suggests that UVA light-induced oxidative stress has a greater contribution in impairing proteins that participate in DNA repair and replication pathways than in inducing direct damage to DNA. Interestingly, the use of antioxidants strongly protected the cells from the damaging effects of UVA-light, justifying the use of antioxidants in sunscreen creams. The hope is that the antioxidants would not only reduce cell killing effects of sunlight, but also reduce mutagenesis and skin cancer risk, by improving NER-mediated removal of the mutagenic photoproducts.

Various proteins linked to DNA repair are targets for oxidation by UVA light (Karran and Brem, 2016), but other genotoxic agents that induce oxidative stress have also been reported to promote inhibition of DNA repair. XPA and XPE proteins (from NER) were shown to be directly affected by the oxidative stress caused by arsenic (Grosskopf *et al.*, 2010; Zhou *et al.*, 2015). Arsenite also damages PARP1, causing inhibition of poly(ADP)-ribosylation and thereby, interfering with BER (Ding *et al.*, 2009). PCNA, a key replication and repair protein is also damaged by singlet oxygen generated from UVA activated photosensitizer generating an oxidative crosslink between two subunits, involving a histidine residue in the intersubunit domain (Montaner *et al.*, 2007). OGG1, a central glycosylase for 8-oxoG repair (BER) in human cells was inhibited by oxidative stress induced by cadmium (Bravard *et al.*, 2006) or by the inflammatory cytokine TNF-alpha (Morreall *et al.*, 2015). Moreover, OGG1 was inhibited by 6-thioguanine (6-TG) activated by UVA light (Gueranger *et al.*, 2014). Partial inactivation of MUTYH, Ku70 and Ku80 proteins due to 6-TG and UVA light was shown to also compromise BER and NHEJ repair activities (Gueranger *et al.*, 2014). UVA and photosensitizers also oxidized XRCC3 protein, impairing homologous recombination (Girard *et al.*, 2013). Most of these cases of protein oxidation have been related to the oxidation of cysteines, sensitizing the cells to DNA damage by affecting the DNA repair pathways. Of special interest is the oxidation of RPA in human cells caused by photosensitizers and UVA light (Gueranger *et al.*, 2014; Guven *et al.*, 2015). RPA is the main protein that stabilizes single strand DNA (ssDNA) and has central roles in DNA repair processes (such as NER and BER) and replication of undamaged and damaged templates (Cimprich and Cortez, 2008; Lukas *et al.*, 2011; Jones and Petermann, 2012). Surprisingly, it has been demonstrated that RPA is the main limiting factor for NER, after UVA irradiation with photosensitizers. This was shown by measuring NER capacity *in vitro* with extracts from cells that were treated with 6-TG and UVA light, where supplementing or overexpression of RPA recovered NER activity (Gueranger *et al.*, 2014; Guven *et al.*, 2015). In fact, oxidation of RPA by UVA and photosensitizer seems responsible for a decrease in the cell’s ability to remove CPD, 6-4PP and 8-oxoG, thus affecting badly NER and BER (Guven *et al.*, 2015).

**Figure 2:**
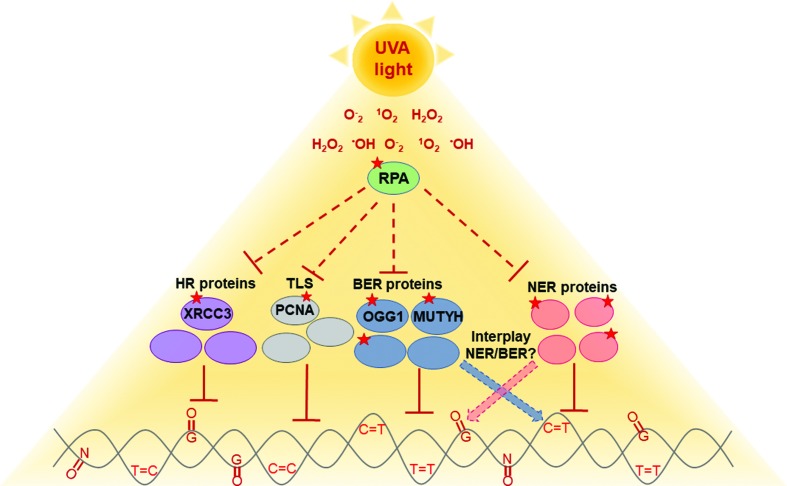
UVA light induces mixture of photoproducts and oxidized base damage in the DNA, as well as, ROS production. As result of UVA-induced ROS production, protein oxidation has been gaining attention because it can also damage DNA repair proteins, which acts on both NER and BER. Thus, UVA irradiation causes decreased repair capacity of target lesions of both pathways, which may be the result of RPA oxidation, or the oxidation of other NER and BER proteins. RPA impairment may further compromise other DNA repair or tolerance pathways, such as homologous recombination (HR) and translesion synthesis (TLS). Red stars represent proteins known to be target of oxidative stress.

RPA is also an important player in DNA damage responses (DDR), where it accumulates and stabilizes ssDNA, recruiting checkpoint and other DNA repair proteins to the damage site. RPA in ssDNA is also a signal to PCNA ubiquitination which is the main regulator of the TLS pathway (Ghosal and Chen, 2013). Therefore, oxidative stress not only impairs RPA protein, but it can also destabilize the signaling of pathways that are necessary for the removal of and/or tolerance to different types of DNA damage (Figure 2). Disruption of such important pathways that control DNA damage may be an aggravating factor for people with DNA repair deficiencies such as XP. As cells from these patients are more sensitive to DNA damage, their use has been proposed to better understand the effects of UVA-light in human cells (Schuch *et al.*, 2017). In fact, evidence that protein oxidation due to UVA-light may aggravate XP cells’ phenotype has been obtained from NER and pol eta deficient cells (Cortat *et al.*, 2013; Moreno *et al.*, 2019a). Pol eta (XP-V) deficient cells are able to repair bulky DNA lesions such as CPDs induced by UVC light but have impaired NER when these lesions are induced by UVA light, probably due to protein oxidation. The use of antioxidants protected UVA-irradiated cells, improved CPD removal, as well as the ability of these cells to replicate their damaged DNA (Moreno *et al.*, 2019a). Moreover, the lack of pol eta and other TLS proteins has been reported to impair NER due to the recruitment of RPA to TLS site (Auclair *et al.*, 2010; Tsaalbi-Shtylik *et al.*, 2014), and the limiting effect of this protein may be even stronger in conditions of oxidative stress. Finally, BER proteins have not been yet evaluated in this context, but as target of oxidation, this pathway may also be affected by UVA-light. Therefore, protein oxidation is not only an important cancer risk factor for XP patients, but also for the normal population.

### NER and BER interactome analysis

**Figure 3:**
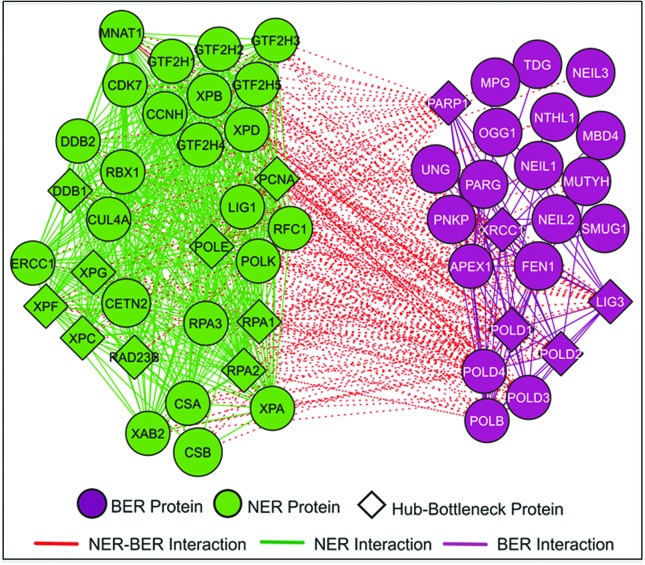
PPI Network depicting the major players in both NER and BER. High connectivity between the two pathways can be observed in the network, which is composed of 55 proteins (32 from NER, 23 from BER) and 734 edges. The parameters used in the STRING software for the *Homo sapiens* organism, were: (i) medium confidence score of 0.4; (ii) “expression”, “databases”, “*neighborhood*” and “co-expression” interaction sources enabled; (iii) only queried proteins on the 1^st^ shell; and (iv) no interactions on the 2^nd^ shell. The centralities analyzed were node degree and betweenness, where 16 HB were identified.

To further understand the interplay between the NER and BER pathways, and scrutinize the underlying interaction between their associated proteins, we conducted a systems biology analysis. We listed the proteins that play major roles in both processes, including all their divisions (i.e., TC and GG-NER, and monofunctional and bifunctional DNA N-glycosylases for BER). By employing the metasearch engine STRING 11 (https://string-db.org/) (Szklarczyk *et al.*, 2017), we prospected a protein-protein interaction (PPI) network composed of 32 proteins related to NER and 23 associated to BER. The initial network created in STRING was used as input in the software Cytoscape 3.6.1 for manipulation (Shannon *et al.*, 2003). Additionally, aiming to identify the most topologically relevant nodes in the PPI network, we employed the Cytoscape plug-in CentiScaPe 2.2 (Scardoni *et al.*, 2009) for degree and betweenness centrality analysis. Degree calculates the number of interactions of each node, and nodes with above average degree values are called “hubs”. Betweenness calculates the number of shortest paths that go through each node, and these nodes with above average scores are named “bottlenecks”. Hence, the hub-bottlenecks (HB) nodes are the most topologically relevant nodes and retain critical regulatory roles within the cell, being classified as “bridges” between biological processes and key molecular modulators (Yu *et al.*, 2007; Pang *et al.*, 2016). Figure 3 portrays the crosstalk between NER and BER and Table S1, lists all interactions between the NER and BER processes from the network.

Clearly, the PPI network reveals a very high number of interactions (229) among the proteins of the two pathways. Nevertheless, the NER pathway appears with more intragroup connections than the BER process (432 and 73 connections, respectively). This maybe due to the fact that BER have many DNA damage recognizing proteins that act more independently one from the other.

Some proteins did not show any inter-pathway connection, been only associated with their own repair mechanisms, these proteins were: (i) NEIL1, NEIL2, MBD4, SMUG1 and PNKP for BER; but none for NER. These aspects should be taken lightly. The lack of intergroup interaction does not necessarily mean that they do not participate in other repair mechanisms, only that they are not the major inter-pathway integrators. For example, MBD4 is a multidomain protein with four different protein regions with a role in the apoptotic pathway, while TDG is related to epigenetic modulation of embryonic development (Sjolund *et al.*, 2013). The proper interpretation is that those proteins, when it comes to the interplay between NER and BER, are not the major bridges between the two pathways.

With the exception of the DNA polymerase δ subunits, PARP1 has one of the highest number of connections in the NER group, interacting with almost all proteins and being one of the top nodes in terms of intergroup connectivity (29 out of 39 connections), besides being an important HB. The HBs present in the network were DDB1, XPC, XPF, XPG, RAD23B, RPA1-2, POLE and PCNA for NER, and POLD1-2, LIG3, XRCC1 and PARP1 for BER. It is expected that proteins such as POLE, POLD1-2, LIG3 and PCNA appear as HB, due to their broad and pivotal role in genome replication and maintenance. Additionally, the appearance of RPA and DDB1 as HB is also not surprising, taking into consideration that both proteins are widely associated to different DNA repair pathways, cell cycle, replication, among others (Fanning *et al.*, 2006; Zou *et al.*, 2006; Iovine *et al.*, 2011). Most of the other HB proteins that show strong intergroup connections are discussed above for their participation on both BER and NER, but it is interesting to mention the high level of connections (on both pathways) of LIG3 (22 intergroup interactions out of a total of 33 connections) an XRCC1 (23 intergroup out of 35 connections).

## Conclusions

DNA repair pathways have been classified as they were discovered, and, in general, they are considered to perform independent and different functions. This is the case for NER and BER, which are normally related to the removal of bulky or modified base lesions, respectively. On the other hand, agents that cause DNA damage, typically generate different types of lesions, that may require different DNA repair pathways to maintain genome stability. Although many efforts have been made to understand the interplay between NER and BER proteins, we know relatively little of these connections. We presented current data on the action of NER proteins on oxidatively damaged DNA, and the role of BER proteins in the protection to agents that form bulky DNA lesions. There is no consensus on the participation of specific proteins in this interplay. The oxidation of repair proteins, mainly RPA, promotes impairment of both NER and BER, adding a new level of complexity to this intricate question. By evaluating the known interactions among NER and BER proteins, the interactome, presented in Figure 3 tells us that there are many connections that are still poorly understood and how they affect these two pathways remains to be elucidated. Understanding this dynamic interplay at specific types of lesions, might prove important in unraveling the underlying mechanisms of carcinogenesis, aging, and neurodegeneration.

## Supplementary material

The following online material is available for this article:

Table S1 All BER X NER inteactions found in the interactome.
