# Supplementary material for: Cooperation and interplay between base and nucleotide excision repair pathways: From DNA lesions to proteins
Source: Genet Mol Biol. 2020 Mar 2;43(1 Suppl 1):e20190104. doi: 10.1590/1678-4685-GMB-2019-0104 (PMC7198027; doi:10.1590/1678-4685-GMB-2019-0104)
Supplement: Supplementary file 1 [file 1415-4757-GMB-43-1-s1-e20190104-suppl1.pdf]

Supplementary material to
"Cooperation and interplay between base and nucleotide excision repair pathways: From DNA lesions to proteins"

Table S1 - All BER X NER interactions found in the interactome.

Source protein	Interacting partners
RAD23B	DDB2<br>RPA3<br>ERCC1<br>GTF2H2<br>CDK7<br>DDB1<br>GTF2H4<br>RBX1<br>RPA1<br>GTF2H1<br>XPC<br>MPG<br>CSA<br>XPG<br>XPF<br>XPB
LIG1	RBX1<br>GTF2H4<br>CDK7<br>RPA3<br>RPA1<br>POLK<br>APEX1
GTF2H1	GTF2H4<br>CDK7<br>DDB2<br>RPA3<br>LIG1<br>POLK<br>RBX1<br>XRCC1<br>ERCC1<br>RPA1<br>CSA<br>POLD3

Source protein	Interacting partners
XPA	RPA3<br>CETN2<br>GTF2H4<br>RBX1<br>PARP1<br>GTF2H2<br>CDK7<br>POLE<br>POLK<br>XAB2<br>RAD23B<br>DDB1<br>DDB2<br>RPA1<br>XPC<br>RPA2<br>GTF2H1<br>ERCC1<br>CSB<br>CSA<br>XPB<br>XPG<br>XPF<br>POLD4<br>POLD3
CDK7	POLK<br>ERCC1<br>RBX1<br>RPA1<br>RPA3
XRCC1	RBX1<br>CDK7<br>GTF2H4<br>RPA3<br>POLK<br>RPA1<br>APEX1
LIG3	PARP1<br>GTF2H4<br>GTF2H2<br>DDB1<br>RBX1<br>GTF2H1<br>CDK7<br>RPA3<br>CUL4A<br>POLK<br>POLE<br>RPA2

Source protein	Interacting partners
	XAB2<br>RPA1<br>XRCC1<br>CSA<br>NEIL2<br>XPB<br>PNKP<br>FEN1<br>NEIL1<br>POLB<br>POLD4<br>POLD3
RPA1	RPA3<br>RBX1<br>POLK<br>ERCC1<br>APEX1
XAB2	RPA3<br>ERCC1<br>RBX1<br>POLK<br>RPA1<br>GTF2H2<br>LIG1<br>CDK7<br>POLE<br>GTF2H1<br>GTF2H4<br>DDB1<br>XRCC1<br>XPB<br>XPG<br>XPF<br>CSA<br>POLD4<br>POLD3
RPA3	RBX1<br>ERCC1<br>APEX1
RPA2	RPA3<br>RPA1<br>POLK<br>XAB2<br>RAD23B<br>GTF2H2<br>RBX1<br>GTF2H4<br>DDB1<br>POLE

Source protein	Interacting partners
	XRCC1<br>ERCC1<br>DDB2<br>GTF2H1<br>LIG1<br>CETN2<br>XPC<br>PARP1<br>CDK7<br>UNG<br>POLB<br>XPF<br>CSA<br>XPB<br>XPG<br>APEX1<br>FEN1<br>POLD4<br>POLD3
POLE	GTF2H4<br>DDB2<br>CDK7<br>RBX1<br>GTF2H1<br>RPA3<br>XRCC1<br>ERCC1<br>POLK<br>DDB1<br>RPA1<br>LIG1<br>CSA<br>XPB<br>POLB<br>APEX1<br>XPF<br>FEN1<br>POLD4<br>POLD3

Source protein	Interacting partners
GTF2H5	GTF2H3<br>GTF2H4<br>XRCC1<br>LIG1<br>XPA<br>PCNA<br>CUL4A<br>POLK<br>XPC<br>DDB1<br>PARP1<br>DDB2<br>RFC1<br>LIG3<br>XAB2<br>RPA1<br>POLE<br>RPA2<br>ERCC1<br>CETN2<br>RAD23B<br>RBX1<br>RPA3<br>CDK7<br>XPD<br>GTF2H2<br>GTF2H1<br>XPF<br>CSA<br>XPG<br>XPB<br>POLD4<br>POLD3<br>POLD1

Source protein	Interacting partners
GTF2H3	GTF2H4<br>XPD<br>GTF2H2<br>GTF2H1<br>XPA<br>PARP1<br>XRCC1<br>RAD23B<br>LIG1<br>RBX1<br>POLK<br>CUL4A<br>RFC1<br>LIG3<br>POLE<br>ERCC1<br>XAB2<br>DDB1<br>DDB2<br>CETN2<br>PCNA<br>RPA3<br>RPA2<br>RPA1 XPC<br>CDK7<br>XPG<br>CSA<br>XPF<br>XPB<br>POLD4<br>POLD3<br>POLD1
XPD	GTF2H2<br>GTF2H1<br>RPA3<br>DDB2<br>CETN2<br>RPA2<br>CUL4A<br>RBX1<br>ERCC1<br>RAD23B<br>LIG3<br>RPA1<br>PARP1<br>XRCC1<br>POLK XPC<br>POLE<br>XAB2<br>DDB1

Source protein	Interacting partners
	XPA<br>LIG1<br>PCNA<br>RFC1<br>GTF2H4<br>CDK7<br>POLD4<br>POLD3
DDB2	CDK7<br>ERCC1<br>RPA3<br>POLK<br>RPA1<br>RBX1
GTF2H2	GTF2H1<br>GTF2H4<br>RPA3<br>XRCC1<br>ERCC1<br>POLK<br>LIG1<br>DDB2<br>RPA1<br>RBX1<br>POLE<br>XPC<br>DDB1<br>CDK7<br>XPF<br>CSA<br>XPB<br>POLD4<br>POLD3
RFC1	DDB2<br>GTF2H4<br>XPA<br>POLK<br>GTF2H1<br>XRCC1<br>RBX1<br>CUL4A<br>DDB1<br>GTF2H2<br>RPA3<br>XAB2<br>ERCC1<br>CDK7<br>PARP1<br>RPA2

Source protein	Interacting partners
	LIG3<br>RPA1<br>POLE<br>LIG1<br>PCNA<br>POLB<br>CSA<br>XPB<br>APEX1<br>XPF<br>XPG<br>FEN1<br>POLD4<br>POLD3
GTF2H4	RPA3<br>ERCC1<br>POLK<br>RPA1<br>RBX1<br>DDB2<br>CDK7
XPC	RPA3<br>RBX1<br>ERCC1<br>CDK7<br>GTF2H4<br>DDB2<br>RPA1<br>GTF2H1<br>POLB<br>CSA
CETN2	PARP1<br>GTF2H1<br>DDB2<br>CDK7<br>ERCC1<br>RPA1<br>GTF2H4<br>DDB1<br>RPA3<br>GTF2H2<br>RBX1<br>RAD23B<br>XPC<br>POLB<br>CSA<br>XPB<br>XPG<br>XPF

Source protein	Interacting partners
PCNA	LIG1<br>RAD23B<br>DDB2<br>GTF2H1<br>ERCC1<br>XAB2<br>GTF2H4<br>GTF2H2<br>CDK7<br>DDB1<br>XPA<br>RBX1<br>XRCC1<br>CUL4A<br>PARP1<br>LIG3<br>POLK<br>RPA3<br>POLE<br>RPA2<br>RPA1<br>NEIL3<br>CSB<br>MUTYH<br>XPB<br>CSA<br>XPF<br>UNG<br>APEX1<br>XPG<br>POLB<br>FEN1<br>POLD4<br>POLD3
RBX1	ERCC1
DDB1	XRCC1<br>GTF2H1<br>POLK<br>RPA3<br>CDK7<br>RPA1<br>GTF2H4<br>ERCC1<br>LIG1<br>XPC<br>RBX1<br>DDB2<br>APEX1<br>UNG<br>XPB

Source protein	Interacting partners
	CSA<br>POLD3
CUL4A	RPA3<br>XPA<br>GTF2H2<br>POLE<br>CDK7 XPC<br>RPA2<br>PARP1<br>CETN2<br>XRCC1<br>ERCC1<br>GTF2H1<br>RPA1<br>POLK<br>XAB2<br>GTF2H4<br>RAD23B<br>LIG1<br>DDB2<br>RBX1<br>DDB1<br>XPF<br>XPB<br>XPG<br>CSA<br>POLD4<br>POLD3
POLK	RPA3<br>RBX1<br>ERCC1
PARP1	XRCC1<br>GTF2H2<br>GTF2H1<br>CDK7<br>POLE<br>RBX1<br>GTF2H4<br>POLK<br>ERCC1<br>DDB1<br>RAD23B<br>RPA3<br>RPA1<br>DDB2<br>XPC<br>CSB<br>XPF<br>XPB

Source protein	Interacting partners
	XPG<br>APEX1<br>FEN1<br>POLB<br>POLD4<br>POLD3
MUTYH	POLE<br>RPA1<br>APEX1
CSB	OGG1<br>XPF<br>XPG
SMUG1	APEX1
MBD4	APEX1
MPG	APEX1
NEIL2	XRCC1<br>POLB
TDG	PCNA<br>APEX1
OGG1	XPC<br>NEIL2<br>APEX1
NTHL1	APEX1
NEIL1	NEIL2<br>OGG1<br>PNKP<br>XRCC1<br>POLB
PNKP	NEIL2<br>POLB<br>XRCC1
PARG	PCNA<br>POLB<br>APEX1<br>FEN1<br>PARP1
POLB	RPA1<br>RPA3<br>LIG1<br>APEX1<br>XRCC1<br>POLD3
CSA	POLK<br>ERCC1

Source protein	Interacting partners
	XRCC1<br>RPA1<br>GTF2H4<br>RPA3<br>LIG1<br>CDK7<br>DDB2<br>RBX1<br>POLD3
XPF	XRCC1<br>NTHL1<br>DDB2<br>RPA3<br>CSA<br>RBX1<br>DDB1<br>GTF2H4<br>CDK7<br>XPB<br>POLK<br>RPA1<br>GTF2H1<br>XPC<br>ERCC1<br>POLD3
XPG	NTHL1<br>CSA<br>RBX1<br>DDB2<br>XPC<br>RPA3<br>DDB1<br>POLK<br>POLE<br>RPA1<br>XPF<br>GTF2H4<br>CDK7<br>ERCC1<br>XPB<br>GTF2H1<br>GTF2H2<br>POLD4<br>POLD3
XPB	XRCC1<br>RPA3<br>ERCC1<br>CSA<br>DDB2

Source protein	Interacting partners
	RBX1<br>POLK<br>RPA1<br>LIG1<br>XPC<br>GTF2H1<br>CDK7<br>GTF2H4<br>POLD3
FEN1	UNG<br>ERCC1<br>XRCC1<br>POLB<br>APEX1<br>RPA1<br>RPA3<br>LIG1<br>POLD3
POLD2	FEN1<br>DDB2<br>XPA<br>GTF2H5<br>GTF2H3<br>CUL4A<br>GTF2H2<br>RBX1<br>GTF2H1<br>ERCC1<br>DDB1<br>CDK7<br>POLK<br>GTF2H4<br>XPD<br>RPA3<br>XRCC1<br>XAB2<br>PARP1<br>LIG3<br>RFC1<br>RPA2<br>LIG1<br>POLE<br>RPA1<br>PCNA<br>UNG<br>MUTYH<br>CSA<br>XPB<br>POLB<br>XPF

Source protein	Interacting partners
	APEX1<br>XPG<br>POLD4<br>POLD3<br>POLD1
POLD4	DDB2<br>CDK7<br>LIG1<br>XRCC1<br>POLK<br>GTF2H4<br>RPA3<br>GTF2H1<br>DDB1<br>RBX1<br>ERCC1<br>RPA1<br>POLB<br>APEX1<br>FEN1<br>XPB<br>CSA<br>XPF<br>POLD3
POLD3	APEX1<br>GTF2H4<br>ERCC1<br>CDK7<br>DDB2<br>RBX1<br>RPA3<br>XRCC1<br>RPA1<br>LIG1<br>POLK

Source protein	Interacting partners
POLD1	RPA1<br>POLD3<br>PCNA<br>CETN2<br>RBX1<br>GTF2H2<br>CUL4A<br>DDB2<br>RAD23B<br>GTF2H1<br>CDK7<br>GTF2H4<br>XPD<br>MUTYH<br>DDB1<br>CSB<br>PARP1<br>CSA<br>XPA<br>XPB<br>XPC<br>POLB<br>ERCC1<br>APEX1<br>XAB2<br>XPG<br>LIG3<br>XPF<br>FEN1<br>XRCC1<br>POLK<br>RFC1<br>RPA2<br>LIG1<br>POLD4<br>POLE<br>RPA3
XAB2	CCNH<br>MNAT1
PARP1	MNAT1<br>CCNH
RAD23B	CCNH<br>MNAT1
XPA	MNAT1<br>CCNH
POLD4	CCNH<br>MNAT1

Source protein	Interacting partners
XRCC1	MNAT1<br>CCNH
LIG3	CCNH<br>MNAT1
POLE	CCNH<br>MNAT1
DDB2	CCNH
RFC1	MNAT1<br>CCNH
XPC	MNAT1<br>CCNH
CETN2	MNAT1<br>CCNH
DDB1	MNAT1<br>CCNH
CUL4A	CCNH<br>MNAT1
POLD1	MNAT1<br>CCNH
POLD2	MNAT1<br>CCNH
POLD3	MNAT1<br>CCNH
GTF2H4	CCNH
RPA2	MNAT1<br>CCNH
MNAT1	DDB2<br>POLK<br>ERCC1<br>RPA1<br>RPA3<br>RBX1<br>GTF2H4<br>CCNH<br>CDK7
LIG1	MNAT1<br>CCNH
PCNA	CCNH<br>MNAT1
GTF2H5	MNAT1<br>CCNH
GTF2H1	MNAT1<br>CCNH

Source protein	Interacting partners
GTF2H3	MNAT1<br>CCNH
CCNH	POLK<br>ERCC1<br>RBX1<br>RPA1<br>RPA3<br>CDK7
GTF2H2	MNAT1<br>CCNH
